# Histomorphometric Evaluation of Peri-Implant Bone Response to Intravenous Administration of Zoledronate (Zometa^®^) in an Osteoporotic Rat Model

**DOI:** 10.3390/ma13225248

**Published:** 2020-11-20

**Authors:** Amani M. Basudan, Marwa Y. Shaheen, Abdurahman A. Niazy, Jeroen J. J. P. van den Beucken, John A. Jansen, Hamdan S. Alghamdi

**Affiliations:** 1 Department of Periodontics and Community Dentistry, College of Dentistry, King Saud University, Riyadh 11451, Saudi Arabia; abasudan@ksu.edu.sa (A.M.B.); mashaheen@ksu.edu.sa (M.Y.S.); 2 Department of Oral Medicine and Diagnostic Sciences, College of Dentistry, King Saud University, Riyadh 11451, Saudi Arabia; aaniazy@ksu.edu.sa; 3 Department of Dentistry-Biomaterials, Radboudumc, 6500HB Nijmegen, The Netherlands; Jeroen.vandenBeucken@radboudumc.nl (J.J.J.P.v.d.B.); John.Jansen@radboudumc.nl (J.A.J.)

**Keywords:** dental implants, osseointegration, osteoporosis, zoledronate, animal model

## Abstract

We evaluated the response to peri-implant bone placed in the femoral condyle of osteoporotic rats, following intravenous zoledronate (ZOL) treatment in three settings: pre-implantation (ZOL-Pre), post-implantation (ZOL-Post), and pre- + post-implantation (ZOL-Pre+Post). Twenty-four female Wistar rats were ovariectomized (OVX). After 12 weeks, the rats received titanium implants in the right femoral condyle. ZOL (0.04 mg/kg, weekly) was administered to six rats 4 weeks pre-implantation and was stopped at implant placement. To another six rats, ZOL was given post-implantation and continued for 6 weeks. Additional six rats received ZOL treatment pre- and post-implantation. Control animals received weekly saline intravenous injections. At 6 weeks post-implantation, samples were retrieved for histological evaluation of the percentage of bone area (%BA) and of the percentage of bone-to-implant contact (%BIC). BA% for ZOL-Pre (29.6% ± 9.0%) and ZOL-Post (27.9% ± 5.6%) rats were significantly increased compared to that of the controls (17.3% ± 3.9%, *p* < 0.05). In contrast, ZOL-Pre+Post rats (20.4% ± 5.0%) showed similar BA% compared to Saline controls (*p* = 0.731). BIC% revealed a significant increase for ZOL-Post (65.8% ± 16.9%) and ZOL-Pre+Post (68.3% ± 10.0%) rats compared with that of Saline controls (43.3% ± 9.6%, *p* < 0.05), while ZOL-Pre rats (55.6% ± 19%) showed a BIC% comparable to that of Saline controls (*p* = 0.408). Our results suggest that receiving intravenous ZOL treatment before or after implant placement enhances peri-implant bone responses in terms of bone area. However, the effect of different ZOL treatment regimens on BIC% was found to be inconclusive.

## 1. Introduction

The worldwide use of dental implants is still increasing, as these fixtures offer many advantages above the more conventional prosthetic reconstruction of lost teeth [1]. This is also because of improved long-term prognosis thanks to innovations in implant design [2]. Overall, the success of dental implants is attributed to their ability to integrate well in living bone (i.e., osseointegration) [3]. However, bone–implant integration can be challenging in compromised conditions, like in the absence of sufficient bone mass and density. Such a condition is frequently diagnosed in older adults and is called osteopenia [4]. Osteopenia is an initial condition that can develop into osteoporosis and is characterized by a reduction in bone density below a standard score compared to healthy young adults [5]. Especially, women are at higher risk for osteopenia as a consequence of menopausal estrogen deficiency [6]. Osteopenia makes bones weaker, which results in a higher risk of fractures and delayed bone healing [7]. Consequently, it can be hypothesized that dental implants installed in patients suffering from osteopenia are more prone to failure. Currently, there is no consensus regarding the effect of osteoporosis on the survival rate of dental implants [8,9]. Clinically, some studies observed significantly higher implant failure in patients with osteoporosis [10,11], while other studies could not associate osteoporosis with increased implant loss [12,13]. An explanation for this discrepancy can be the used selection criteria for inclusion or exclusion of patients in the various clinical studies, the wide variation in implant types, and the administration of different drugs [9]. Therefore, it is recommended that standardized experiments are performed to elaborate further on this topic and clarify this matter. The present study is part of a series of experiments, which were designed to fill in the lack of knowledge about the relationship between osteoporosis and bone–dental implant response.

The pharmaceutical treatment of osteoporosis includes the use of a wide range of drugs (e.g., bisphosphonates, statins, drugs for estrogen replacement therapy (ERT), calcitonin, parathyroid hormone, and calcium supplements) as well as various routes of administration (i.e., oral or systemic) [14]. Besides being used to decrease fracture risk in osteoporotic patients, these drugs have also been suggested to support bone formation around dental implants [14,15]. The data of these preclinical studies generally indicated that the application of systemic bisphosphonate treatment has a positive effect on implant osseointegration [16]. Bisphosphonates (BPs) are a class of drugs capable of mediating bone metabolism and reducing bone turnover [17]. BPs exhibit antiresorptive effects by reducing osteoclastic recruitment and activity [18]. Moreover, BPs have been reported to stimulate the function of osteoblasts [19,20]. A recent systematic review by Gelazius et al. examined the effect of bisphosphonates on dental implant placement procedure, and the results showed no significant implant success rate difference in intravenously and orally medicated groups. Moreover, patients treated with intravenous bisphosphonates seemed to have a higher chance of developing implant-related osteonecrosis of the jaw, and implant placement in patients treated intraorally could be considered safe with precautions [21]. One of the most potent BPs is zoledronate (ZOL), which is characterized by long-term retention in bone tissues compared to other BPs [22,23]. ZOL is primarily administrated intravenously (IV) to avoid gastrointestinal adverse effects associated with oral administration of BPs [24,25]. ZOL has been shown to favor the bone–implant response [26,27,28]. It is also well documented that ZOL has possible side effects, as it may cause initial influenza-like illness associated with the first infusion. Renal failure has been noted in patients with cancer after repetitive high-dose ZOL infusions. Moreover, osteonecrosis of the jaw after tooth extraction has been recorded as a result of the systemic administration of ZOL [29]. However, the effect of the timing of drug administration, i.e., before and/or after placing dental implants, is not clear [14]. Considering all this, we hypothesized that an initial treatment targeting an osteopenic bone condition before implant installation would be more favorable for implant–bone integration compared to a treatment started after implant placement.

Therefore, the purpose of the present study was to evaluate the bone response to titanium implants placed in osteoporotic rats treated with intravenous ZOL at three different times (pre-implantation, post-implantation, and pre- and post-implantation) compared to non-treated controls.

## 2. Materials and Methods

### 2.1. Ovariectomy Rat Model

All animal experiments in the present study were in accordance to the guidelines (national and international) for animal care and conformed to the ARRIVE guidelines. The Animal Ethics Committee at King Saud University, Saudi Arabia, approved the present study (No. 4/67/389683). Twenty-four female Wistar rats (weight of ~250 g) were used. The animals were housed in standardized rat cages (4–5 animals per cage) maintained in a laboratory environment with controlled temperature (22–24 °C) and humidity (45–55%) and 12 hourly light and dark cycles. All animals had ad libitum access to a standard rat chow diet and water. All rats were subjected to bilateral ovariectomy (OVX) procedures under general anesthesia (GA) to induce an osteoporotic condition as previously described [30].

### 2.2. Sample Number Estimation

The power analysis of sample size was done using the formula (n1 = n2 = n3 = 1 + 2 × c(s/d)^2^). Standard deviation (s) was 12.5, effect size (d) was 15, C was 7.85 (resulting from 1-β = 0.8 and α = 0.05). The final result was 24 rats (one implant per rat).

### 2.3. Implantation Procedures and Study Groups

At 12 weeks post-ovariectomy, a sterile condition was applied for the implantation surgery. A single intraperitoneal injection of 0.2 mg/kg xylazine (Chanazine, Chanelle Pharmaceutical, Galway, Ireland) and 0.5 mg/kg ketamine hydrochloride (Ketamine, Pharmazeutische Praparate, Gießen, Germany) was used for general anesthesia. Then, the right rat’s leg was shaved and disinfected using 10% Povidone–iodine (Alphadin, MedicScience Life Care Pvt. Ltd., Haryana, India). An incision was made over the knee joint to expose the femoral condyle. Then, we drilled a small hole (1.5 mm in diameter and 8 mm in depth) using surgical burs and a rotary handpiece along with saline irrigation. Then, a commercially available mini-implant (Unitek™ TADs, 3M Oral Care, St Paul, MN, USA, diameter, 1.8 mm, length, 8 mm) was placed in the prepared hole (1 implant per rat). Finally, the skin was sutured with 4-0 resorbable sutures (VICRYL^®^ Polyglactin 910, Ethicon, Johnson & Johnson, New Brunswick, NJ, USA). For the anti-osteoporotic treatment, a ZOL injection (Zometa^®^ 4 mg, Novartis, Basel, Switzerland) was prepared and administered intravenously via the tail vein, as described previously [26]. For the experiment, rats were divided into four equal groups (n = 6 rats per group):

1. ZOL-Pre: weekly ZOL administration (0.04 mg/kg body weight) in the 4 weeks prior to implant placement [31].
2. ZOL-Post: weekly ZOL administration in the 6 weeks from implant placement until the end of the study (=6 weeks) [14].
3. ZOL-Pre+Post: weekly ZOL administration from 4 weeks prior to implant placement until the end of the study (=10 weeks).
4. Saline: weekly intravenous injection with 1 mL saline from 4 weeks prior to implant placement until the end of the study; this group was considered the non-treated control.

### 2.4. Animal Euthanasia and Specimen Retrieval

All rats were euthanized with CO_2_ after 6 weeks of healing. The bone specimens were retrieved and fixed in 10% formalin for 2 days. Then, the samples were kept in 70% ethanol for histological preparation.

### 2.5. Histological and Histomorphometric Examination

**Figure 1:**
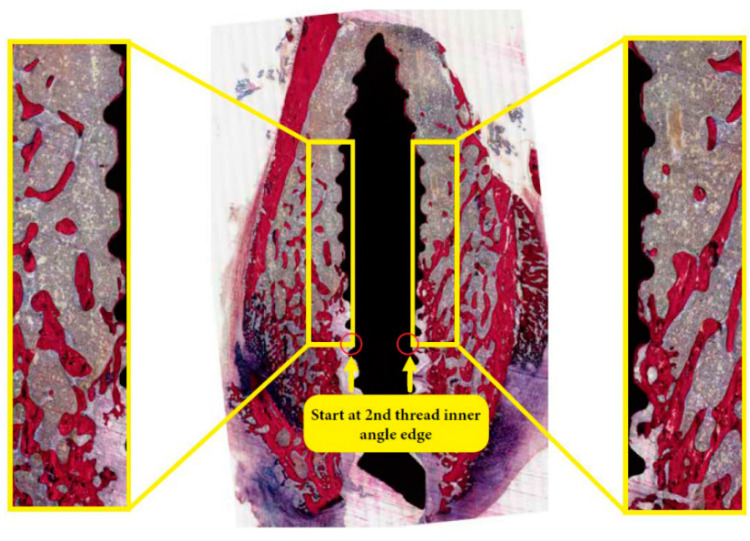
Sequence of representative histological sections (stained with methylene blue and basic fuchsin) showing the region of interest (ROI) for the measurement of bone area (BA%) and bone-to-implant contact (BIC%): from the left and right sides of the implant (yellow box).

First, the bone samples were dehydrated in ethyl alcohol from 70% to 100%, then embedded in poly(methylmethacrylate) (pMMA) resin. Thereafter, longitudinal sections (10 µm thick) were prepared using a microtome and stained with methylene blue and basic fuchsin. A light microscope (Aperio ImageScope, Leica Biosystems, Buffalo Grove, IL, USA) was used for their observation. Histologic observations were performed by two examiners (AB & JJ). Using the Aperio ScanScope (Aperio ImageScope, Leica Biosystems, Buffalo Grove, IL, USA) image extraction tool, individual histology images were extracted in 25% jpg format. An image analysis software (IMAGE-J 1.4, National Institute of Health, Bethesda, MD, USA) was used to perform blinded histomorphometric measurements for three histological sections per implant (at ×20 objective magnification). The color hue and saturation of bone tissues were selected and standardized to red, while the other tissues were in yellow. Then, bone area (BA%) was measured in a region of interest (ROI) on both sides of the implant, i.e., a rectangular box (1 mm width, 4 mm length) that started at the 2nd top implant thread (Figure 1). Bone-to-implant contact (BIC%) was assessed by manually measuring the relative length of bone tissue in direct contact with the implant. The measurements from both sides of the implant in three different sections were averaged and used for statistical analysis.

### 2.6. Statistical Analysis

SPSS Statistical Program (Version 26, IBM, USA) was used to perform the descriptive statistics of BA% and BIC%, as mean and standard deviation (SD). Comparisons between test and saline groups were performed using one-way analysis of variance (ANOVA) with Dunnett post-hoc test, with statistical significance of *p* < 0.05.

## 3. Results

### 3.1. Rat Model

Animals in all groups showed no clinical complications or infections post-implantation. Table 1 summarizes the number of implants placed and retrieved and the numbers of sections used for histomorphometric analyses.

### 3.2. Histological Evaluation

**Figure 2:**
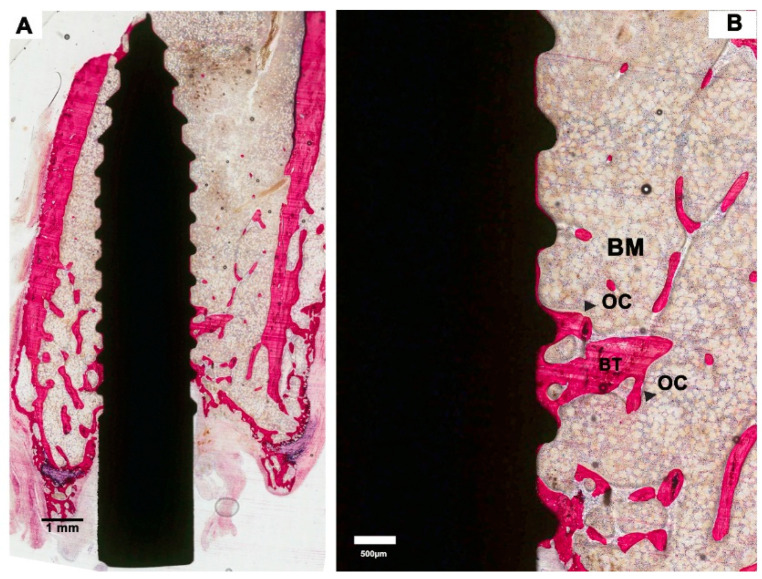
Histological sections of an implant in the saline group (control). (**A**). Low-magnification image showing a limited amount of bone formation. (**B**). Representative histological images at higher magnification showing osteoclast-like cells (OC) on the surface of the bone trabeculae (BT). Bone marrow (BM) was present between the bone trabeculae and in the areas where bone was lacking. Between the tips of the screw threads, a thin layer of bone is visible on the implant surface.

Saline controls: light microscopical examination of the histological sections revealed that the bone of the femoral condyle had an osteopenic appearance, which was characterized by the presence of a limited amount of bone trabeculae as well as a wide spacing between the bone trabeculae (Figure 2A). Bone marrow was present between the bone trabeculae and in the areas where bone was completely lacking. The bone looked very mature and lacked substantial remodeling activity. Only occasionally, osteoclasts were observed, which were in tight contact with the bone trabeculae. Although very limitedly, bone trabeculae did make contact with the implant surface (Figure 2A,B). The majority of the contact sites was at the tip of the screw thread. On the other hand, all implants were covered for a significant part of their surface with a very thin layer of bone (Figure 2B). The bone was in close contact with the implant surface without intervening fibrous tissue layers. Again, the remodeling activity of the deposited bone was very limited, and no active layer of osteoblasts was observed.

**Figure 3:**
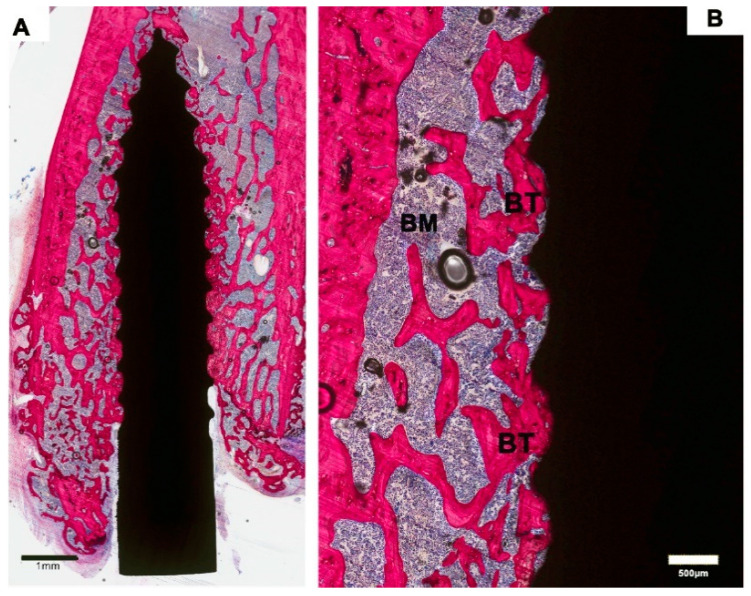
Representative histological images of an implant in the ZOL-Pre study group. (**A**). Low-magnification image showing bone trabeculae surrounding most of the implant. (**B**). Higher magnification revealed that bone was in tight contact with the majority of the implant surface.

**Figure 4:**
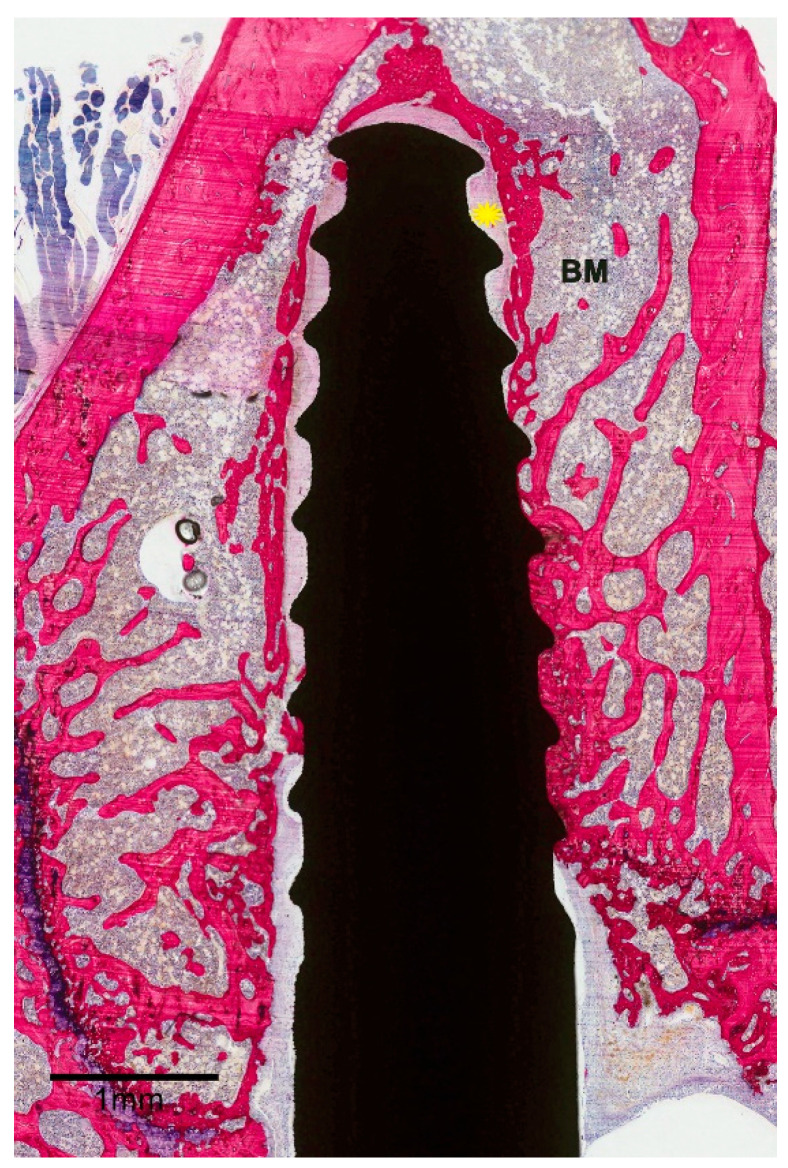
Histological image showing a “failed” implant. The implant was surrounded by a wide space filled with fibrous tissue (yellow star).

ZOL-Pre: examination of the histological sections suggested that the amount of bone in the femoral condyle as well as surrounding the implant was increased compared to Saline controls (Figure 3A). This increase was very evident in three of the six specimens but was more limited in the other three specimens. Strikingly, the remodeling activity of the bone was very low (Figure 3B). Also, the major part of the implant surface was covered with a thin layer of bone, but bone formation was not very active, as no layer of osteoblasts nor osteoid was observed. One of the specimens was considered a failure (Figure 4), as about 75% of the implant was not in direct contact with bone and was surrounded by a wide gap with a width of approximately 0.1–0.2 mm. This gap was filled with fibrous tissue, but no inflammatory reaction was observed.

**Figure 5:**
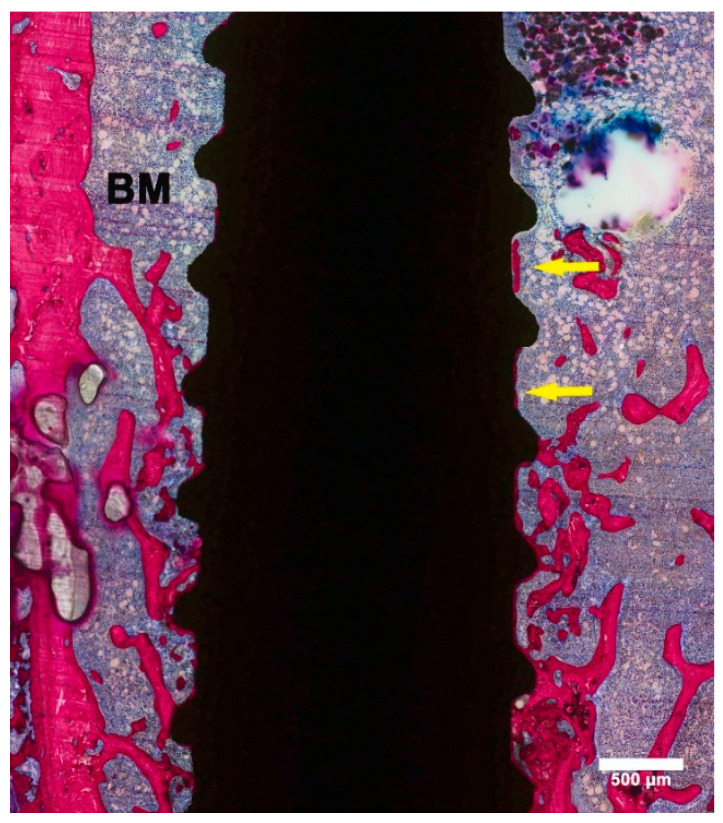
Light micrograph of a specimen of the ZOL-Post study group. Trabecular bone appears in contact with the implant surface, and a thin layer of bone covers part of the implant surface (yellow arrows).

ZOL-Post: a light-microscope assessment indicated features largely similar to those observed in ZOL-Pre rats. The bone amount in the femoral condyle and around the implants was larger compared to that of Saline controls and appeared very similar to that observed in ZOL-Pre rats (Figure 5). Remodeling activity of the bone was not obvious. Trabecular bone was in contact with the implant surface in all retrieved specimens, and a thin layer of bone was present on all implant surfaces (Figure 5).

**Figure 6:**
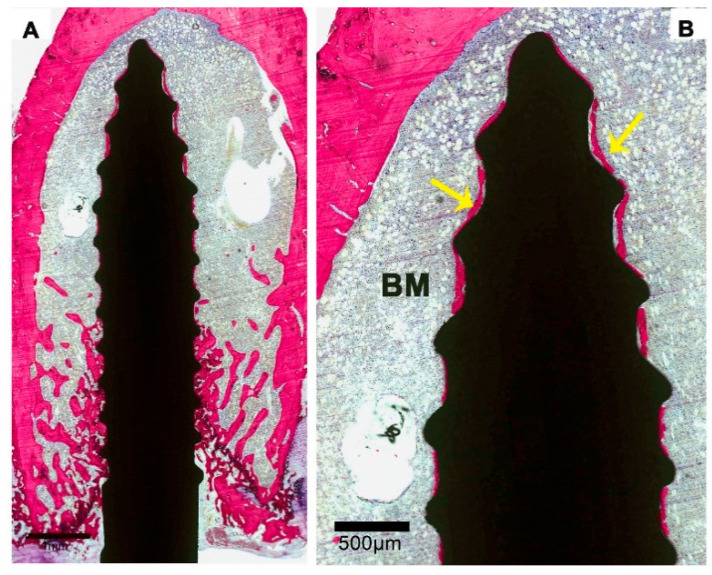
Low- and high-magnification images of a specimen of the ZOL-Pre+Post group. (**A**) Low-magnification image showing that bone formation mainly occurred at the crestal side of the implant and extended about halfway up the implant length. (**B**) High magnification revealed that a thin layer of bone covered the implant surface (yellow arrows).

ZOL-Pre+Post: histological images suggested enhanced bone formation in the femoral condyle. In contrast to ZOL-Pre and ZOL-Post rats, the increase was mainly seen at the crestal side of the implant and extended about halfway up the implant length (Figure 6A). Areas that were not occupied by bone trabeculae were filled with bone marrow. Trabecular bone was in contact with the implant surface, and bone remodeling activity was low. A thin layer of bone was covering major areas of all implants and bone was also deposited in areas where no trabecular bone was present (Figure 6B).

### 3.3. Histomorphometric Evaluation

**Figure 7:**
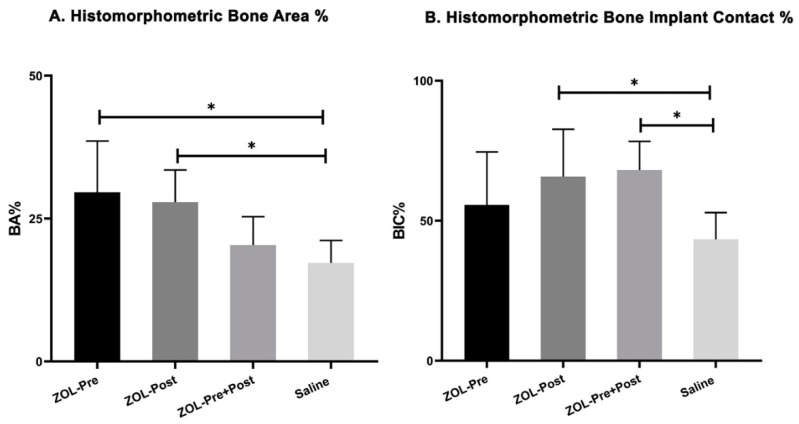
Bar chart with mean and standard deviation for (**A**) BA% and (**B**) BIC% in all study groups. (* indicates *p* < 0.05).

Both BA% and BIC% were determined and are shown in Table 2. One specimen in Saline controls was excluded from the histomorphometric analysis, as the histological sections did deviate too much from a section plane parallel to the longitudinal axis, which made proper measurements impossible. Further, the failed specimen of the ZOL-Pre group was excluded from the measurements. Data showed a mean BA% of 29.6% ± 9.0% for ZOL-Pre, 27.9% ± 5.6% for the ZOL-Post, 20.4% ± 5.0% for ZOL-Pre+Post, and 17.3% ± 3.9% for Saline controls. The results of the repeated measures analysis of variance with Dunnett’s multiple comparison test revealed that BA% for ZOL-Pre and ZOL-Post was significantly higher compared to that of Saline controls (*p* = 0.013 and 0.025, respectively, Figure 7A). In contrast, ZOL-Pre+Post rats showed similar BA% compared to that of Saline controls (*p* = 0.731). BIC% values were 55.6% ± 19.0% for ZOL-Pre, 65.8% ± 16.9% for ZOL-Post, 68.3% ± 10.0% for ZOL-Pre+Post, and 43.3% ± 9.6% for Saline controls. Statistical testing revealed a significant difference for ZOL-Post and ZOL-Pre+Post with respect to Saline controls (*p* = 0.049 and 0.027 resp), while ZOL-Pre showed a BIC% comparable to that of Saline controls (*p* = 0.408, Figure 7B).

## 4. Discussion

In the present study, the peri-implant bone response was compared in OVX rats, which were treated with ZOL (0.04 mg/kg, once a week) via intravenous infusion at three different times: pre-implantation, post-implantation, and pre+post-implantation; rats receiving weekly saline injections served as controls. Both bone area (BA%) and bone–implant contact (BIC%) were histomorphometrically evaluated. In comparison to saline controls, the pre- and post-implantation ZOL treatment showed a significant gain of BA% (*p* < 0.05). Additionally, BIC% data showed a significant difference when comparing post-implantation and pre+post-implantation ZOL-treated rats to saline controls (*p* < 0.05).

One of the treatment approaches for osteopenia/osteoporosis is the systemic administration of ZOL [24,25,32,33]. ZOL has been shown to (1) effectively suppress the recruitment and function of osteoclastic cells that cause bone resorption [22,32] and (2) have a positive effect on bone formation by stimulating osteoblasts, in comparison to other BPs [34]. Moreover, several studies reported that ZOl has a greater impact and stronger effects on osseointegration than other BPs [28,35]. Therefore, ZOL is considered a potent bisphosphonate, whose effectiveness has been confirmed in several clinical trials involving osteoporotic patients [36]. The recommended administration option of ZOL for treating osteoporosis is by once-yearly intravenous infusion of 5 mg [37].

Although bisphosphonates have a relatively good safety record and are tolerated by the majority of patients, these drugs are reported to be associated with several side effects. For instance, the most notable adverse drug reaction associated with oral bisphosphonates is upper gastrointestinal discomfort, which may include heartburn, indigestion, esophageal erosion, and esophageal ulcer [38]. In order to avoid these reactions, drugs should be administered with a full glass of water in the morning on an empty stomach 30 min prior to a meal or other medications (60 min for ibandronate). Additionally, patients should remain upright for at least 30 min post-dose to prevent esophageal irritation. Osteonecrosis of the jaw is another complication detected in patients receiving prolonged intravenous bisphosphonate therapy, especially ZOL, who undergo invasive dental procedures, such as tooth extractions and implant placement. Physicians should consider either discontinuation or a drug holiday when the risks of use outweigh the benefits [38].

Considering the above-mentioned observations, we intended to examine in the current experimental animal study the effect of ZOL administration on bone formation around dental implants installed in osteoporotic bone. Although a wide variety of animal models is available, ovariectomized rats are well established to closely simulate osteopenia/osteoporotic bone conditions and the response to pharmacological therapy observed in humans [39]. As such, we utilized ovariectomized rats to study whether systemic ZOL administration before and/or after implant placement improves the peri-implant bone response.

Our study design for evaluating the peri-implant bone response in osteoporotic animals is distinct from previous studies, which focused on testing the effect of ZOL administration only post-implantation. Our aim was to test different clinical scenarios, as can occur in patients with postmenopausal osteopenia requiring dental implant installation. For example, August et al. [40] observed in a retrospective manner that postmenopausal women with an osteopenia-like condition in their maxilla showed a significant failure rate (13.6%) of installed dental implants compared to healthy women (6.3%, *p* = 0.039) if they did not receive anti-osteoporotic therapy before implant surgery. Consequently, we decided to investigate the effect of three ZOL administration regimens related to the moment of implant installation. A key aim was to determine whether post-implantation continuation of the administration of ZOL would be more beneficial in comparison with only pre- or post-implantation drug administration. A delay in the delivery of ZOL to the implant site was hypothesized to have less pharmacological benefit than pre-implantation ZOL binding to the target site. In addition, prolonged ZOL administration was supposed to better “treat” the diseased bone at the implant site. In a continuous approach, the preoperative administration of ZOL has to control the unbalanced bone metabolism, while the postoperative administration of ZOL has to enhance bone–implant healing [22].

However, the above-mentioned hypothesized effect of a continuous approach was not proven. In contrast, an improvement in BA% around the implants was observed for the intravenous administration of ZOL to OVX rats for only 4 weeks pre-implantation or only post-implantation. However, no favorable effect was seen for the continued administration, which led to a BA% comparable to that non-treated osteoporotic rats. The beneficial effect of only pre- or post-implantation administration of ZOL on BA% corroborates other studies. For example, Yoshioka at al. [41] demonstrated that systemic administration of ZOL in osteoporotic ovariectomized rats resulted in increased bone formation after 4 weeks. Another study assessed the effect of simultaneous insertion of titanium implants and systemic treatment with ZOL in osteoporotic and sham-operated rabbits [42]. They found that ZOL treatment significantly increased the BA% in the osteoporotic rabbits 3 months after surgery. Additionally, Bobyn et al. [43] examined the effect of an intravenous injection of ZOL (0.1 mg/kg) post-implantation in a dog model and reported that ZOL increased peri-implant bone formation by twofold compared to non-treated controls [43]. Cardemil et al. revealed that a systemic single dose of ZOL in ovariectomized animals improved bone-to-implant contact [31]. Recently, He et al. [29] reviewed the literature and described 10 preclinical studies that confirmed a possible effect of systemic ZOL administration on implant osseointegration. Consequently, the conclusion seems justified that intravenous ZOL can improve implant osseointegration in osteoporotic animals.

On the other hand, the pre+post-implantation ZOL treatment of osteoporotic rats did not favor BA% increase. An explanation for this finding can be related to the applied doses of ZOL. The effect of nitrogen-containing BPs, like ZOL, on osteoclasts is known to be depending on dose and dose frequency [44]. In addition, it has to be remarked that the concentration of ZOL in the plasma after intravenous administration reduces rapidly due to the fast adsorption of the ZOL onto the bone surface [45]. In line with this, the efficacy of ZOL dosage was investigated in a clinical study involving patients suffering from Paget’s disease [46]. The data of this study indicated that doses above 200 g had a reduced therapeutic effect.

As mentioned earlier, the recommended treatment approach for osteoporosis is a once-yearly intravenous infusion of ZOL. In our study, ZOL was administered once weekly for a period of 10 weeks. Therefore, it cannot be excluded that this dosing was too high, resulting in a decreased effect on bone formation. In view of this, it is recommended to determine the alkaline phosphatase (ALP) level in the blood serum of the rats in future studies. This will provide information about the therapeutic effect of ZOL in an osteoporotic condition.

The measurements of BIC% showed a significantly higher BIC% in rats receiving post- and pre+post-implantation ZOL treatment compared to saline controls. These data are inconclusive, and their relevance is not evident. These inconsistent observations can be explained by a low remodeling activity of the trabecular bone surrounding the implant bed in all treatment scenarios. How this interferes with bone formation at the implant interface remains elusive. In combination with the relatively short implantation time, it can be assumed that the bone present at the implant surface was mainly already existing bone and not newly deposited bone. In this condition, BIC% represents just the amount of bone present during implant installation. This explanation is in agreement with other studies, which suggested that a follow-up time of 6 weeks is too short, and more than 8 weeks of observation are necessary to detect a possible effect of pharmacological drugs on the interfacial implant–bone response [29,47].

Additionally, implant location as well seems to have a significant effect on the results. For instance, Cardemil et al. studied the different biological reactions of the tibia and the mandible in rats, both in response to ovariectomy and in response to the ZOL treatment. They reported an increase of BIC in the tibia, whereas the opposite occurred in the mandible [31]. This variation can be attributed to different turnover rates and density in different bones [48].

Finally, a comment has to be made regarding the experimental conditions of the current study. Some authors noticed that a high calcium diet may reverse the condition of osteopenia in ovariectomized animals [49]. In our model, OVX rats were fed a standard diet with a considerable amount of calcium. This was done with the purpose of mimicking the human clinical situation: human osteoporotic patients are not given a specific diet but are recommended to eat healthily and include sufficient amounts of dairy products as well as vitamin D-containing food in their meals daily. Nevertheless, the standard diet may have influenced the effect of ZOL on the osteoporotic-like bone in the present study.

## 5. Conclusions

Under the current experimental conditions, our results suggest that receiving intravenous ZOL treatment before or after implant placement enhances peri-implant bone responses in terms of bone area. However, the effect of different ZOL treatment regimens on BIC% was found to be inconclusive. Further, the following recommendations can be made for future studies dealing with implant installation in osteoporotic animals and co-administration of anti-osteoporotic drugs: (1) allow an implantation time longer than 8 weeks and (2) determine ALP blood serum levels to assess the therapeutic efficacy of the anti-osteoporotic drugs.

## Figures and Tables

**Table 1:** Summary of implants placed and submitted to histological evaluation. ZOL, zoledronate.

Groups	Osteoporotic Condition		
	No. Placed Implants	No. Used Implants	No. Histological Sections
**ZOL-Pre**	6	5 *	15
**ZOL-Post**	6	6 ^#^	18
**ZOL-Pre+Post**	6	6	18
**Saline**	6	5 *	14

* Two implants failed (no osseointegration); ^#^ Three implants had bicortical penetration of cortical layers. ZOL-Pre, ZOL administration prior to implant placement, ZOL-Post, ZOL administration after implant placement, ZOL-Pre+Post, ZOL administration prior and after implant placement (see text for details).

**Table 2:** Mean and standard deviation (SD) for BA% and BIC% in the different study groups.

Study Groups	ZOL-Pre	ZOL-Post	ZOL-Pre+Post	Saline
Bone area (BA%) (Mean ± SD)	29.6 ± 9.0	27.9 ± 5.6	20.4 ± 5.0	17.3 ± 3.9
Bone–implant contact (BIC%) (Mean ± SD)	55.6 ± 19.0	65.8 ± 16.9	68.3 ± 10.0	43.3 ± 9.6
